# Factors affecting addiction severity index (ASI) among clients enrolled in methadone maintenance treatment (MMT) program in Myanmar

**DOI:** 10.1186/s12954-021-00523-2

**Published:** 2021-08-05

**Authors:** Sun Tun, B. Vicknasingam, Darshan Singh

**Affiliations:** 1 Myanmar Medical Association, Yangon, Myanmar; 2 Centre for Drug Research, Universiti Sains Malaysia, George Town, Penang Malaysia

**Keywords:** Methadone maintenance therapy, Opioid addiction, Addiction severity index (ASI), Illicit drug use, Myanmar

## Abstract

**Background:**

Opioid substitution with methadone maintenance treatment (MMT) is shown to reduce illicit opioid use and renew social functioning. Understanding factors that undermine clients’ social functioning during MMT treatment is vital for improving treatment compliance and quality of life.

**Method:**

A total of 210 respondents who were already enrolled in a formal MMT program in Myanmar were recruited from five cities through stratified random sampling for this cross-sectional study. The addiction severity index (ASI) was used to objectively assess respondents social functioning in the last 30 days. Higher ASI scores denote poorer social functioning.

**Result:**

Respondents total ASI scores in the respective domains were: employment (47.4%), alcohol (44.4%), drug use (7.2%), legal (49.2%) and social–family relationship (10.7%). Those reported to have never injected drugs in the last 30 days had lower ASI total scores than those who reported injection drug use (*p* = 0.01). After identifying the differences in ASI total scores, we found there were significant associations in the clients’ hepatitis C status, age category, frequency of heroin injection, quality of life score, marital status, current leisure status with family/friend, current history of injection in the last 30 days, income status, satisfaction with current marital status, as well as reported drug and alcohol use (*p* < 0.05). Stepwise binary logistic regression showed that alcohol and higher frequency of heroin injection were associated with higher ASI scores. Meanwhile, older age, respondents those who had leisure time with family, and satisfied with current marital status had lower ASI scores (*p* < 0.05).

**Conclusion:**

Our results indicate that those enrolled in the MMT program in Myanmar faced many challenges in their daily social functioning. Treatment providers must take heed of these apparent impediment to ensure clients chequered social functioning does not undermine their treatment compliance.

Trial registration: NA

## Background

The United Nations Office on Drugs and Crime (UNODC) estimated that there are about 57.8 million opioid users globally [1]. The UNODC found that almost half of those who injected drugs were living with HIV and one in eight injectors had hepatitis C [2]. Opioid substitution therapy (OST) with methadone maintenance treatment (MMT) is the most promising harm reduction intervention for reducing illicit opioid use [3]. Although MMT is shown to curtail HIV spread and improve health, it is also equally important to evaluate clients social functioning in the MMT program [4]. A systematic review and meta-analysis of methadone treatment report a decrease in the self-reported arrest rate, clients’ drug selling rate, rates of selling sex for drugs and drug-related crime from the baseline to 6 month and 12 months of intervention. Moreover, the rate of employment and proportion of clients reporting a good relationship with their family increased substantially after treatment started [5].

In Myanmar, the MMT programme was first introduced in 2006 primarily to curb HIV spread among injecting drug users. As of 2019, a total of 19,991 people who inject drugs (PWIDs) have enrolled in the MMT program in Myanmar [6]. With the support of public and private sectors who work collectively to mitigate HIV spread, both sectors were also responsible for ensuring the MMT program functions effectively. The National Strategic Plan for HIV/AIDS (2021–2025) plans to ensure that 40% of PWIDs in 2025 are enrolled in the MMT programme. Since clients in the MMT program are bound to face impairments in social functioning, it is vital for treatment providers to identify factors undermining clients social functioning during the MMT treatment including factors affecting the ASI. A qualitative interview on methadone patients in Myanmar found out that lack of an individualized treatment plan made it difficult to maintain the treatment and resulted in poor outcomes [7]. The research conducted in Yangon reported that the infection rates of HIV, HCV and HBV among those enrolled in methadone were high. It showed that their testing and subsequent treatment could be improved by the health education session of the methadone program, which also prevented further transmission to their community and the public. Furthermore, it was also recommended the development of participant-focused and more decentralized services of a methadone programme would prevent clients from leaving of methadone program [8]. Meanwhile, the research on social factor identification of the methadone clients in Myanmar is still a gap in the methadone literature.

Where injecting drug use (principally opioids) was one of the leading causes of HIV transmission, stopping injecting drug use led to a slowing down in the progression of HIV disease in infected subjects. But placing PWIDs on methadone treatment is not a complete solution. If the patients are retained on OST longer will have better outcomes. A high methadone dose, a key determinant of treatment retention with 60 mg or greater, is also associated with improved retention and outcomes. Furthermore, improved outcomes are also associated with provisional treatment of primary and specialist medical treatment, psychosocial support services and a range of ancillary services [9]. Treatment retention at 6 months was reported as high, uniformly averaging 70% across the countries in that Lawrinson study. Meanwhile, recent data from Myanmar also reported 68% retention in the program data [10].

In the prospective mixed methods cohort study in Imphal, India (a border area with Myanmar), the provision of OST caused significant reductions in needle sharing, in drug use over the previous 4 weeks, in property crime, anxiety, depression and suicidal thoughts when compared to the baseline and 12 months after treatment. Additionally, meaningful improvements in physical health, mental health, the quality of family relationships, and participation in family events, employment and hope for the future were reported from this cohort study. The study also included dropped-out clients from the methadone program with enrich understanding of the lived experience of OST clients. In the review of mental health and addiction services, the qualitative aspect of a family-centred approach to care was seen as an essential component, rather than client centre approach, especially in an Asian culture [11]. Another longitudinal research reported from Poland also acknowledged that methadone patients significantly changed their coherence; their lives are more understandable, predictable and meaningful, with the realization of all developmental tasks. However, their cognitive emotional regulation strategies and intensity of psychopathological symptoms did not change significantly after 6 months of treatment [12].

Given that clients in the MMT program are susceptible to experiencing social impairments, this study aims to generally define MMT client social functioning and identify factors undermining their social functioning in MMT treatment in Myanmar.

## Methods

### Study design, respondents and location

A total of 210 clients receiving methadone treatment participated in this cross-sectional study. All the respondents for this study were recruited through stratified random sampling from five cities (Yangon, Mandalay, Lashio, Kawlin and Mohnyin) in Myanmar where the MMT program is formally implemented. Each site from all of the States and Regions was included to ensure different client and provider characteristics were reflected. Data collection area sampling was done by randomizing the eligible sites based on the sample size requirements. A randomized plan for sampling site was created on www.randomization.com, and the data were used for selecting the study sites for Kachin, Shan, Sagaing, Mandalay and Yangon. Since the respondents were vulnerable and marginalized in nature, patients who gave the consent to answer the survey questionnaires and to give urine specimen were recruited randomly by the drop-in-centre service providers/resource people from the eligible candidate list (i.e. at least 6 month treatment) until the proposed sample size of 42 respondents was reached from each site. Personal interview and urine sample collection procedures were done in a private room at the drop in centres/ restaurant by the researchers at each site who were working on harm reduction projects. The addiction severity index-Lite (ASI) [13] with structured questionnaires was used to objectively determine respondents social functioning.

### Inclusion and exclusion criteria

Inclusion criteria for the study include: (1) must be above 18 years of age, (2) presently enrolled in MMT programme and tested positive for methadone in urine and (3) must have a minimum of six-month MMT history. We included the exclusion criteria that those who hesitate to give their informed consent and those experiencing significant health and psychological problems. However, there were no clients excluded with these criteria in this study during the actual recruitment process and it can be assumed that the respondents’ social functioning status was covered.

### Measures

All the study data were collected from May to July 2017. All the surveys were conducted through face-to-face interviews by two trained researchers. A semi-structured questionnaire was used to collect respondents’ socio-demographic characteristics, quality of life information, HIV risk behaviours, previous drug use history, and history of infectious disease and methadone treatment experiences. We also used the validated addiction severity index-Lite (ASI) [14], WHOQOL-BREF [15] and time line follow back (TLFB survey) [16] questionnaires to collect respondents drug use history. TLFB was added for accurate recall of the recent drug use status (previous 7 days) of the respondents, in addition to the ASI questionnaire.

For the addiction severity index (ASI) scores, 5 out of 7 composite scores were collected and analysed for employment, alcohol use, drug use, legal status and family/social composite scores. Medical and psychiatric composites were excluded. Each raw composite score was calculated and transformed to a 0–100 scale using the formula shown below:

$${\text{Transformed}}\;{\text{scale}} = \frac{{\left( {{\text{Actual}}\;{\text{raw}}\;{\text{score}} - {\text{lowest}}\;{\text{possible}}\;{\text{raw}}\;{\text{score}}} \right)}}{{{\text{Possible}}\;{\text{raw}}\;{\text{score}}\;{\text{range}}}} \times 100$$

where “Actual raw score” is the values achieved through the summation of responses from each composite score, “lowest possible raw score” is the lowest possible value that could occur through the summation (this value would be 4 for all facets of employment) and “Possible raw score range” is the difference between the maximum possible raw score and the lowest possible raw score.

### Urinalysis

All the respondents were screened for methadone use prior to their participation in the study. Rapid test kits were used to confirm the respondents’ illicit drug use status for morphine, cannabis, methamphetamine, amphetamine and benzodiazepine use. Rapid urine drug test kits were ordered from BioTesT (China) [17]. Rapid test strips were used after immersing in the urine specimen for 10–15 s, and then, the results could be read after 5 min. The cut-off values established by the manufacturer of the urine tests were methadone above 300 ng/ml, morphine above 300 ng/ml, cannabinoids (THC) for marijuana above 50 ng/ml, methamphetamine above 1000 ng/ml, amphetamine above 1000 ng/ml and benzodiazepine above 300 ng/ml.

### ASI composite score calculation

#### Composite score for employment status

ASI composite score for employment status was included questions on driver’s license and car availability for their use, paid work in the past 30 days, income from work in the past 30 days. Those responses were combined and transformed to 0–100 scale for comparison among the responses.

Our ASI calculation for the ASI score (Employment) and other ASI domain calculation technique was referred per Composite Scores Manual [14].

ASI calculation for employment score, there are 4 specific questions under the employment status.

A. Do you have a valid driver’s license?
B. Do you have an automobile available for your use?
C. How many days were you paid for working in the past 30?
D. How much did you receive from employment (new income) in the past 30 days?

*Step 1* Composite score for employment status is calculated with the formula “1.000 − (A/4 + B/4 + C/120 + log D/36)” and we derive raw composite score from employment questions.

*Step 2* The ASI raw score for “Employment domain” comes with the range from − 0.87 to + 1.69 and resulted total range 2.56. As different range of ASI scores for different domains varies (e.g. alcohol (− 2.08 to + 1.17, range 3.25, etc.), it is difficult to interpret with unequal total ranges. So, we applied the transformed scale formula to compare different ranges of raw score of different domains to “0–100” scale, which can be easily seen the severity in percent score for all domains.

$${\text{Transformed}}\;{\text{scale}} = \frac{{\left( {{\text{Actual}}\;{\text{raw}}\;{\text{score}} - {\text{lowest}}\;{\text{possible}}\;{\text{raw}}\;{\text{score}}} \right)}}{{{\text{Possible}}\;{\text{raw}}\;{\text{score}}\;{\text{range}}}} \times 100$$

This similar approach of calculation for the rest of the domains is referred as described by the Composite Scores Manual, and results are described in percent in Table 2 [with employment (47.4%), alcohol (44.4%), drug use (7.2%), legal (49.2%) and social–family relationship (10.7%)].

#### Composite score for alcohol use

The composite score for alcohol use was derived from the responses to the questions of the number of days of any alcohol use/ intoxication in the past 30 days, the number of days troubled or bothered by any alcohol problems, how it was troubled in the past 30 days, the importance of treatment for alcohol problems and money spent during the past 30 days on alcohol. Alcohol-related responses were combined and transformed to 0–100 scale for comparison among the responses.

#### Composite score for drug use

The composite score for drug use was derived from the responses to the questions of number of days use of drugs (heroin, methadone, other opiates/analgesics, barbiturates, other sedatives, cocaine, amphetamines, cannabis, hallucinogens), number of days used more than one drug, number of days drug use problems were experienced in the past 30 days, how the patient was troubled in the past 30 days and how important they viewed treating their drug problems. Drug use-related responses were combined and transformed to 0–100 scale for comparison among the responses.

#### Composite score for legal status

The composite score for legal status was derived from the responses to the questions on status of presently awaiting charges, trial, or sentencing, number of days engaged in illegal activity for profit in past 30 days and the amount of money received from illegal sources in the past 30 days. Legal status-related responses were combined and transformed to 0–100 scale for comparison among the responses.

#### Composite score for family/social status

The composite score for family/social status was derived from the responses to the questions on satisfaction with current marital situation, serious conflicts with their family in the past 30 days, family problems which troubled or bothered the patient in the past 30 days and how important they thought treatment or counselling was for their family problems. Responses were combined and transformed to 0–100 scale for comparison among the responses.

#### Composite score for medical status and psychiatric status

The composite scores for medical status and psychiatric status were not included. Those respondents who couldn’t answer the survey questions due to medical and psychiatric problem were excluded from the client selection criteria. So, these two sessions in the ASI were omitted.

### Statistical analysis

#### Use of statistical tests

The study data were analysed with Stata14.0 software. The responses were summarized and respective composite scores, and total scores were calculated. Chi-square test and Fisher’s exact test were used for identifying association of the ASI score differences between categorical variables. Independent t-tests were used to compare different types of respondent characteristics for continuous variables. For examining the differences between mean ASI scores of the interested parameters, independent t-tests were used for analysis. Due to the limitation of the data availability on the interested vulnerable population, effect size indexes (e.g. Cohan’s *d*) were not taken into considered for the estimation of sample size requirement and its analysis. Missing or incomplete entries were not counted in the formula calculation to achieve a composite score or average ASI score and to have a reliable and consistent calculation. A higher ASI score reflects a poorer functioning situation, and lower ASI scores reflect better functioning of the clients. Binary logistic regression was used for identifying the predictors to the interested outcome “ASI total score”. Stepwise binary logistic regression analysis was done to recheck the significant regression output with the intended output by adjusting the confounding associated variables. All outcomes were set with statistically significance at *p* < 0.05 with two-tailed results.

### Ethical consideration

Ethical approval for the study was obtained from the Human Ethics and Research Committee of Universiti Sains Malaysia (No: USM/ JEPeM/16080269) (University of Science, Malaysia) and Department of Medical Research (No: Ethics/DMR/2017/057), Ministry of Health and Sports, Myanmar [18].

## Results

### Socio-demographic characteristics

Respondents’ socio-demographic characteristics are shown in Table 1. Respondent average methadone dose in this study was 83 mg/day (with a range of between 20 and 300 mg/day). Meanwhile, 132 (63.46%) received a daily methadone dose of less than and equal 80 mg, while 76 (36.54%) had more than 80 mg. The respondents mean duration of methadone treatment history in this study was 28 months (2.4 years). Eighty-three percent (173/210) had one episode of methadone treatment history, while the rest had more than one episode of treatment history. A total of 210 respondents were recruited, 42 from each city in Kawlin (Sagaing), Lashio (Shan), Mandalay (Mandalay), Mohnyin (Kachin), Yangon (Yangon).

**Table 1:** Demographic characteristics of methadone respondents

Variable	Frequency (n and %)	Mean (SD)	Range
*Age (years)*		33.35 (8.85)	20–76
Less than and equal 35 years	129 (61.4%)		
more than 35 years	81 (38.6%)		
*Gender*			
Male	207 (98.6%)		
Female	3 (1.4%)		
BMI (body mass index)	206 (98%)	20.52 (3.38)	14.03–33.39
Less than 18.5 (underweight)	61 (29.61%)		
Between 18.5 and 25 (normal)	123 (59.71%)		
More than 25 (overweight)	22 (10.68%)		
*Working as outreach or peer*			
Yes	29 (13.81%)		
No	181 (86.19%)		
*Education (years)*			
No formal education	6 (2.86%)		
Primary	42 (20%)		
Secondary (Middle + High School)	117 (55.71%)		
College- University	45 (21.43%)		
*Marital status*			
Married	84 (40.58%)		
Separated/divorce/widowed	27 (13.04%)		
Single	96 (46.38%)		
*Living style in recent 3 year period*			
Nuclear family style	72 (34.45%)		
With parents	91 (43.54%)		
Extended family	41 (19.62%)		
Alone	5 (2.39%)		
*Employment (usual/last)*			
Employed	192 (91.43%)		
Un-employed currently (includes disable, student)	18 (8.57%)		

### Scores on addiction severity index

To reflect the severity index of the clients, ASI scores were calculated as shown in Table 2. Some ASI composite questions were not answered, and some are not applicable for the respondents so are not meaningful to interpret from incomplete answers. This causes the varying frequency for each composite scores as described in the table. As there are no data for ASI for medical and psychiatry, these composite scores are not mentioned in the ASI composite score table.

**Table 2:** Table showing ASI scores (transformed on 0–100 scale)

Variable	Frequency (n and %)	Mean (SD)
ASI total score	210 (100%)	84.66 (43.30)
ASI average score	210 (100%)	23.97 (10.49)
*ASI composite scores*		
ASI for employment	207 (98.6%)	47.38 (19.15)
ASI for alcohol use	64 (30.5%)	44.39 (21.12)
ASI for drug use	210 (100%)	7.2 (13.39)
ASI for legal status	28 (13.3%)	49.21 (25.44)
ASI for family/social status	210 (100%)	10.72 (12.70)

From the calculated ASI total scores derived from different domains, mean score differences with the respondents’ characteristics are analysed in Table 3 [19].

**Table 3:** Table showing differences of mean ASI total score with characteristics of the respondents

Demographic characters	Sub groups	Number (n)	Mean ASI total score	*p* value
Age	Younger and equal 35 years	129	88	0.0247**
	Older than 35 years	81	80	
BMI (body mass index)	Less than mean BMI (20.5)	122	88	0.2183
	More than mean BMI	84	80	
Antiretrovials (ART)	No	142	84	0.6534
	On treatment	68	87	
Education	Up to primary	48	85	0.9893
	More than primary	162	85	
Job status	Unemployed	24	80	0.0000 ***
	Employed	183	118	
Current peer/ outreach worker	No	181	87	0.0556
	Peer/outreach	29	70	
Marital status	Currently married	84	70	0.0000***
	Single/separated	123	95	
Income	Low	131	97	0.0000***
	High	78	65	
Current marital status satisfaction	Not satisfied	20	108	0.0098**
	Satisfied	190	82	
*WHO Quality of life (QOL) total score*	Low	88	98	0.0002***
	High	121	75	
Physical QOL score	Low	53	99	0.0062 **
	High	156	80	
Psychological QOL score	Low	41	90	0.3739
	High	168	83	
Social QOL score	Low	71	93	0.0584
	High	138	81	
Environmental QOL score	Low	54	98	0.0111 **
	High	155	80	
*Current leisure status satisfaction*	Not satisfied	30	106	0.0032**
	Satisfied	180	81	
Current leisure status with family	Not satisfied	120	97	0.0000***
	Satisfied	90	68	
Current leisure status with friend	Not satisfied	135	79	0.0200**
	Satisfied	75	94	
Current leisure status alone	Not satisfied	160	80	0.0059**
	Satisfied	50	99	
*Infection history*				
HIV status (No vs Yes)	Not infected	126	82	0.4194
	Infected	74	87	
Hepatitis C status	Not infected	77	78	0.0162**
	Infected	71	95	
Hepatitis B status	Not infected	166	83	0.4711
	Infected	15	94	
Tuberculosis treatment history	No history	147	87	0.2669
	Treated history	54	79	
Sexually transmitted infection history	No history	164	84	0.7533
	Infected history	45	87	
*Abuse encountered within 30 days*	Not experienced	183	83	0.3155
	Experienced	26	93	
Psychological abuse	Not experienced	184	83	0.3703
	Experienced	22	92	
Physical abuse	Not experienced	204	85	
	Experienced	1	93	
Sexual abuse	Not experienced	205	84	
	Experienced	1	62	
*Methadone services*				
Methadone dose categories	Less than and equal 80 mg	132	87	0.4118
	More than 80 mg	76	82	
Methadone duration	Less than and equal 2.4 year	120	85	0.8608
	More than 2.4 year	89	84	
*Methadone treat frequency*	First time treatment	173	84	0.5357
	More than one time	35	89	
*Dose* less than and equal 80 mg	First time treatment	109	86	0.7544
	More than one time	22	89	
More than 80 mg	First time treatment	63	80	0.5339
	More than one time	13	88	
*Duration* less than and equal 2.4 years	First time treatment	95	85	
	More than one time	24	82	0.7389
More than 2.4 years	First time treatment	78	82	0.1795
	More than one time	11	102	

Independent t-test *p* value: **significance < 0.05, ***significance < 0.001

The ASI total score was calculated by totalling the transformed score of each ASI domains for an individual. A high ASI total score was defined as a score above the mean score for the ASI total score of all eligible respondents.

From mean ASI score difference analysis, higher addiction severity index total scores (*p* < 0.05) were highly associated with methadone clients in the younger age group, currently employed, single or separated, low income, clients who were not satisfied with current marital status, marital status as single/ separated, who had less quality of life (Physical and environmental domains), clients not satisfied with current leisure status and clients with hepatitis C infection. However, there were no significant differences of ASI total scores and subscores between different categories of methadone dose (*p* > 0.05). Meanwhile, there were significant differences of ASI total scores on employment and alcohol use. These scores were significantly higher among clients with longer treatment on methadone (*p* < 0.05). ASI score differences with the illicit drug use situation are further analysed in Table 4.

**Table 4:** Differences of mean ASI total score with illicit drug use situation of respondents

Differences in ASI score with illicit drug use situation	Sub groups	Number (n)	Mean ASI total score	p value
*Urine illicit drug findings*				
Urine morphine	Absent	93	85	0.8683
	Present	117	84	
Urine THC	Absent	185	83	0.1104
	Present	25	98	
Urine methamphetamine	Absent	158	85	0.6610
	Present	52	82	
Urine amphetamine	Absent	191	86	0.2132
	Present	19	73	
Urine benzodiazepine	Absent	138	83	0.3846
	Present	72	88	
*Reported drug use status*				
Last heroin injection within 30 days	No	93	75	0.0030**
	Yes	116	93	
Frequency of injection per month	No or few inj: (mean = 7)	162	80	0.0011***
	Higher	47	103	
Needle sharing within 30 days	Not shared	99	92	0.0160**
	Shared	4	150	
Life time sharing of needle and syringes	Not shared	106	79	0.0478**
	Shared	103	91	
Drug and alcohol	Not used	47	60	0.0000***
	Used	162	92	
Alcohol	Not used	144	71	0.0000***
	Used	66	115	
Heroin	Not used	117	78	0.0073**
	Used	93	94	
Morphine	Not used	205	84	0.0508**
	Used	5	122	
Benzodiazepine	Not used	187	82	0.0133**
	Used	22	106	
Barbiturate	Not used	206	84	0.6641
	Used	4	94	
Antidepressant	Not used	206	83	0.0000***
	Used	4	173	
*Cocaine (Not used = 210)*				
Amphetamine	Not used	156	81	0.0646
	Used	54	94	
THC	Not used	186	82	0.0162**
	Used	24	105	
Ecstasy	Not used	207	84	0.3084
	Used	3	110	
Inhalants	Not used	133	80	0.0391**
	Used	76	93	
More than one drug	Not used	50	66	0.0005***
	Used	143	90	

Independent t-test *p* value: **significance < 0.05, ***significance < 0.001

### Urinalysis finding

Results from the urine drug screening show 117 (55.71%) tested for morphine, 54 (25.71%) for amphetamine and methamphetamine, 25 (11.90%) for THC, a cannabis compound and 72 (34.29%) for benzodiazepine. More than two-thirds tested for only one type of drug, while 92 (43.81%) were identified as poly-drug users.

From additional data analysis, ASI drug use scores were significantly higher among clients who injected heroin in the last 30 days and also reported higher frequency of drug injection (*p* < 0.05). Those reported to have never injected drugs in the last 30 days had lower ASI total scores than those who reported injection drug use (*p* = 0.00). Needle sharing in the last 30 days, as well as needle sharing in their life time, was seen among clients with a high ASI employment score (*p* < 0.05). Those clients who reported alcohol and drug use (alcohol, heroin, benzodiazepine, antidepressant, THC, inhalants) and poly-drug use had higher ASI scores (*p* < 0.05).

After identifying the differences in ASI total scores, factors associated with high ASI scores were explored. There was significant association between higher ASI total scores of methadone clients who had hepatitis C, younger age, higher frequency of heroin injection, respondents with lower quality of life score, marital status as single/ separated, respondents who are not satisfied current leisure status with family/friend/or alone, higher injections per month currently, low-income status, not satisfied with current marital status, reported drug and alcohol use among variables mentioned in the tables. For drug and alcohol use, alcohol, heroin, morphine, benzodiazepine, antidepressant, THC, inhalant and those who use more than one drug were associated with higher ASI total scores (*p* < 0.05). These significant parameters were put for further regression analysis.

### Stepwise binary logistic regression analysis

After considering significant association factors in the model affecting ASI total scores (based on significant parameters in Tables 3, 4), stepwise binary logistic regression was done to identify predictors that were linked with high ASI total scores. Retention in logistic regression was used for predicting client characteristics impacted on the total score of addiction severity index. When checking for multi collinearity, the mean variance inflation factor (vif) was 1.89 and none of the variables has more than 10. The regression model alpha ratio is set at 0.05.

Table 5 shows the result of stepwise binary logistic regression.

**Table 5:** Correlates of high ASI total score among methadone respondents

Variables	Adjusted OR (95% CI)	*p* value
Heroin injection frequency	4.11 (1.37, 12.35)	0.012**
Alcohol	24.47 (6.14, 97.51)	0.000***
Age	0.12 (0.04, 0.37)	0.000***
Leisure with family	0.23 (0.82, 0.63)	0.000***
Satisfaction with marital status	0.12 (0.03, 0.57)	0.008**

Stepwise binary logistic regression, *p* value: **significance < 0.05, ***significance < 0.001

In the analysis of adjusted model of binary logistic regression, potential confounding variables (among associated characteristics) were considered for adjustment and estimated the association of independent variables to the outcome (dependent) variable of total score of addiction severity index of the methadone clients. Alcohol alone had 24 times (aOR 24.47, 95% CI 6.14–97.51, *p* = 0.000) while higher frequency of heroin injection had 4 times (aOR 4.11, 95% CI 1.37–12.35, *p* = 0.012) in contributing high addiction severity score of the clients. Being older than 35 years had 8 times (aOR 0.12, 95% CI 0.05–0.37, *p* = 0.000), and leisure with the family reduced addiction severity by 4 times among methadone clients (aOR 0.12, 95% CI 0.04–0.37, *p* = 0.000). Satisfaction with current marital status also lessened severity by 8 times among methadone clients (aOR 0.12, 95% CI 0.03–0.57, *p* = 0.008).

**Fig. 1:**
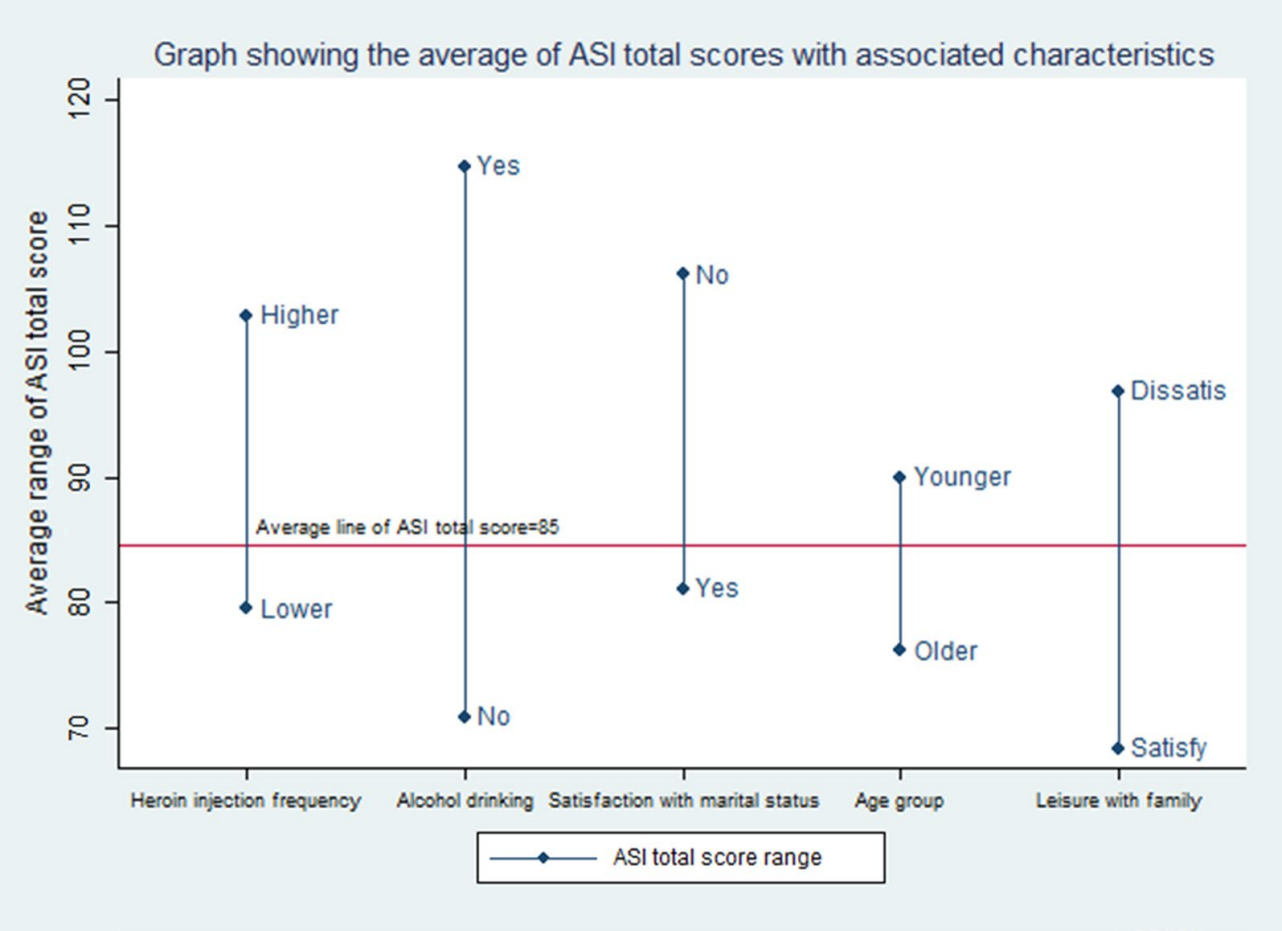
Graph showing the average of ASI total scores with associated characteristics

Significant differences of ASI scores from logistic regression were seen among the client with different characteristics mentioned in Fig. 1.

## Discussion

To the best of our knowledge, this study is among the few available studies that aim to describe MMT clients social functioning in Myanmar. Various studies have shown that clients who are enrolled in the MMT program have better ASI scores or social functioning [20]. We found alcohol and frequency of heroin injection significantly influenced ASI scores. Since the majority of clients were still using drugs, alcohol consumption also highly influenced clients social functioning. High scores on ASI were observed among those reported drug and alcohol use and also among poly-drug users (*p* < 0.05). In another 10-year follow-up study, alcohol highly influenced to the drug use and ASI drug scores were also significant (*p* = 0.0008) [21]. Those who used drugs and alcohol had higher ASI scores in the drug use domain (*p* < 0.05). These findings were in accordance with a previous study [22]. Consequently, an increase in ASI scores for alcohol use was also a strong predictor for dropping out from the MMT program [23].

There were no significant differences in the ASI total scores with methadone dose and duration of treatment. In another longitudinal study, there were significant differences in the ASI drug use domain (*p* = 0.0002) among clients receiving high methadone dose (more than 100 mg) and the ASI family domain (*p* = 0.03) among those receiving moderate doses of methadone (60–100 mg), but significant improvement in the ASI score for drugs in high dose group [24]. A significant finding of Fareed study which contributed to better understanding of these clients was the prospective cohort analysis of the clients. Meanwhile, our current result is from cross-sectional study without eliminating other confounding factors like enzyme inducers in patients taking other treatments (e.g. antiretrovials). This finding was similar to another study showing that those receiving higher methadone doses were (a) HIV infected and (b) HIV and hepatitis C co-infected compared to those without HIV and hepatitis C infections (*p* < 0.0001) as described in the PROTEUS study [25]. This affects methadone dose and has a significant correlation with HCV infection [26]. This indicates that clients with co-infections are prescribed with high methadone dose in Myanmar. Longer treatment duration had significant differences in the ASI employment score (*p* = 0.0029) and ASI alcohol use scores among MMT clients in this study (*p* = 0.0214). This was similar to the findings of a system review of ASI employment score results, reduced at follow-up from baseline with longer duration on methadone (*p* = 0.074) [20]. It was also discussed in similar context where MMT treatment itself exposed the clients with an additional burden which needs with implementation adjustments to include extending clinic hours, offering transportation services and providing take-home doses if feasible [27].

Opioid dependent clients identified by urine had higher ASI scores for employment and drug use (*p* < 0.05). Those who tested for opioid use were more likely to use benzodiazepine (*p* = 0.000). Benzodiazepine use was found to be common among MMT clients [28]. Furthermore, the benzodiazepine use is also a strong predictor for the drop out of the methadone clients within 12 months [29]. Use of benzodiazepine identified by urine in this study was associated with higher ASI scores for family (*p* = 0.0209), low dose methadone clients (*p* = 0.014) and clients who had longer MMT duration (*p* = 0.031).

From this study findings, those who wish to initiate OST treatment should be properly counselled with the correct aims and attitude of taking opioid substitution therapy. Identification of long-term methadone service utilization should be tracked for the changes of treatment parameters. Individualized data tracking can be done, but this has to be done in consideration with medical ethics. Use of the unique client coding system is essential in recording and linking of utilized services with patient treatment episodes and its addiction severity, behaviour changes linked with drug use and treatment. Satisfaction of friend and family support is also crucial in patient social functioning while having effective methadone treatment. This will improve the individual treatment scenario and program planning aspect for each dispensing centres. These points were also highlighted for effective implementation of the OST in the resource poor settings, but not limited to the lack of political will to act, need to change the relevant laws, social and structural discrimination against PWIDs, cost of providing therapy and lack of local evidence [30].

This study has a few limitations. The cross-sectional study design is not possible to prove the parameter differences by the continuous timeline or causal interventions. Another limitation in the sampling is it needs to obtain the total methadone client population for the selected site (city). Due to national data privacy, the random selection was only supported by the harm reduction service providers who had access to the sampling frame. There could be limitation on randomness of selected participants from the selected site.

One additional limitation of this study is the low number of participation from female methadone users which can be due to low female drug use compared to male populations. Furthermore, this may also be due to cultural reasons where fewer female drug users seek treatment compared to male drug users and a lower female response was reflected. The study exclusion criteria also considered the medical and psychological domains as screening tools for patients who couldn’t response well with these factors in participation the survey. Questions on seriousness on the feeling of present legal problems and importance of counselling or referral need for legal problems were not included due to privacy reason.

## Conclusion

Our findings proved that there were significant influential factors that affected clients’ social functioning in the MMT program in Myanmar. There is a need for treatment providers to give more attention to subtle factors affecting clients social functioning. As addiction scores are higher in employment, legal and alcohol, the study highlights a critical intervention need for proper psychosocial counselling and rehabilitation programme with employment supports for methadone clients. Besides, addressing pharmaceutical interventions, creating employment opportunities and enabling social factors like marital and family status satisfaction are also crucial for improving clients social functioning. Further studies are needed to determine clients social functioning in MMT program in Myanmar.

## Data Availability

The [.dta] data used to support the findings of this study are available from the corresponding author upon the approval of the Centre for Drug Research.
